# Prognostic Potential of the Baseline Pan-Immune-Inflammation Value and Neutrophil/Lymphocyte Ratio in Stage I to III Melanoma Patients

**DOI:** 10.3390/cancers14184410

**Published:** 2022-09-11

**Authors:** Thilo Gambichler, Andreas Stang, Rita Mansour, Christina H. Scheel, Celine Nick, Nessr Abu Rached, Jürgen C. Becker, Laura Susok

**Affiliations:** 1 Skin Cancer Center, Department of Dermatology, Venereology and Allergology, Ruhr-University Bochum, 44791 Bochum, Germany; 2 Institute of Medical Informatics, Biometry, and Epidemiology, University Hospital Essen, 45147 Essen, Germany; 3 Department of Dermatology, Translational Skin Cancer Research, German Cancer Consortium (DKTK) Partner Site Essen/Düsseldorf, University Duisburg-Essen, 45147 Essen, Germany; 4 Deutsches Krebsforschungszentrum (DKFZ), 69120 Heidelberg, Germany

**Keywords:** cutaneous melanoma, pan-immune-inflammation value, neutrophil/lymphocyte ratio, biomarkers

## Abstract

**Simple Summary:**

Unlike the neutrophil/lymphocyte ratio (NLR), more complex complete blood count (CBC)-based systemic immune-inflammation cancer biomarkers have recently been proposed, such as the pan-immune-inflammation value (PIV). We aimed to assess both NLR and PIV in cutaneous melanoma (CM) patients. Briefly, we found that, although the higher PIV and NLR values appear to be associated with survival in the crude analysis, adjustment for potential confounders, in particular age and tumor thickness, reduced the strength of association between PIV and NLR on survival substantially. PIV as well as NLR were positively correlated with age and tumor thickness which are important independent predictors for CM relapse and CM-specific death. Both CBC-based parameters appear to be confounded by age and tumor thickness and probably have no potential to further improve the prediction of survival of stage I to III CM patients beyond standard prognostic factors.

**Abstract:**

Prognostic biomarkers derived from complete blood count (CBC) have received marked interest as an indirect measure of the inflammatory pressure in cancers such as metastatic melanoma. Here, we evaluated the novel pan-immune-inflammation value (PIV) and the frequently assessed neutrophil/lymphocyte ratio (NLR) in a large cohort of patients with cutaneous melanoma (CM) without distant metastases (stages I to III). PIV and NLR were calculated at CM diagnosis. Healthy controls were also included. We used the Kaplan–Meier method to estimate crude survival probabilities and used Cox proportional hazards regression for multiple adjustment of hazard ratios. We observed that higher PIV (HR: 1.72, 95% CI 1.14 to 2.58 and HR: 1.696, 95% CI 1.029 to 2.795, respectively) and NLR (HR: 1.70, 95% CI 1.10 to 2.62) values were associated with CM relapse and CM-specific death in the crude analysis. However, when adjusting for potential confounders, in particular age and tumor thickness, the total effect of PIV and NLR on CM-relapse-free (HR: 1.28, 95% CI 0.83 to 1.98 and HR: 1.26, 95% CI 0.80 to 1.98, respectively) and CM-specific survival (HR: 1.36, 95% CI 0.80 to 2.30 and HR: 1.37, 95% CI 0.80 to 2.33, respectively) was substantially reduced. However, both PIV and NLR were positively correlated with age and tumor thickness, which are important independent predictors for CM relapse and CM-specific death. In conclusion, in stage I to III CM patients PIV as well as NLR appear to be confounded by age and tumor thickness and probably have no potential to further improve the prediction of survival of stage I to III CM patients beyond standard prognostic factors.

## 1. Introduction

The incidence of melanoma is increasing worldwide, with more than 55,000 deaths yearly [1,2,3]. The impairment of acute inflammatory responses against carcinogens triggered by the innate immune system creates a predisposition for tumor development and progression. Involvement of smoldering chronic inflammation has been demonstrated in every step of carcinogenesis, including cancer development, progression, and metastasis. Considering the instrumental role of inflammation in cancer, measuring the levels of different types of inflammation could serve as prognostic biomarkers, although data on appropriate parameters and means of quantification remain relatively scarce [4,5,6].

To date, many immune-inflammation-based biomarkers, e.g., count of neutrophils or lymphocytes, neutrophil/lymphocyte ratio (NLR), and platelet/lymphocyte ratio have been proposed as prognostic predictors in a variety of cancers, including melanoma [6,7,8,9,10,11]. Moreover, a novel and more complex blood-based biomarker represents the pan-immune-inflammation value (PIV) [12], which has also been investigated in skin cancer and other malignancies with varying prognostic capacity [12,13,14,15,16].

This study aimed to assess the PIV and NLR in control subjects and a reasonable sample of patients with cutaneous melanoma (CM) stages I to III.

## 2. Methods

### 2.1. Patients

This mono-center investigation was performed at the Department of Dermatology, Ruhr-University Bochum, Germany. All CM patients who had undergone sentinel lymph node (SLN) biopsy between 2001 and 2015 including complete blood count (CBC) data at initial diagnosis were considered. Next, we reviewed the patient files for sufficient data with respect to gender, age, CBC data, Breslow tumor thickness, CM ulceration, and follow-up information. All patients with complete data were included in the following analysis.

The diagnosis of CM was performed by full primary excision and histopathological examination. Vertical tumor thickness of 1 mm or more was an indication for SLN biopsy (SLNB). SLNB was also considered for tumors with: vertical tumor thickness 0.75–1 mm, ulceration, high mitotic index, and age < 40 years. As in our previous study [14], the presence of macro-metastases lymph nodes and distant metastases was assessed by physical examination and investigations, including sonography, computed tomography, and magnetic resonance imaging [17]. Based on the established clinical practices at that time, patients with micro-metastases in the SLN usually underwent complete lymph node dissection. Adjuvant low-dose interferon alfa-2a therapy (Roferon; Roche Pharma AG, Grenzach-Wyhlen, Germany) was carried out in patients with tumor thickness ≥ 1.5 mm and without evidence of SLN micro-metastasis. In patients with positive SLN, adjuvant high-dose interferon alfa-2b (Intron; MSD, Munich, Germany) was usually performed. Follow-up was performed in line with national and international guidelines: specifically, patients with primary CM smaller than 1 mm tumor thickness were seen for follow-up every 6 months, whereas patients with thicker primary melanomas received a follow-up every 3 months, which included lymph node ultrasound and determination of serum S100B and lactate dehydrogenase. Every 6 months, stage III patients additionally underwent whole-body imaging [2]. Healthy controls were also included for the comparison of PIV and NLR.

### 2.2. Laboratory Parameters

We calculated the PIV as follows: [neutrophils (10^3^/mm^3^) × platelets (10^3^/mm^3^) × monocytes (10^3^/mm^3^)]/lymphocytes (10^3^/mm^3^) [12]. NLR was calculated as follows: absolute neutrophils (10^3^/mm^3^)/lymphocytes (10^3^/mm^3^). We determined PIV and NLR at the time of initial diagnosis.

### 2.3. Statistics

Using the continuous distribution of PIV and NLR, we identified three groups of equal size using tertiles. The ranks of thirds for PIV were ≤219.73, >219.73 to 393.73, and >393.73. The ranks for NLR were ≤2.02, >2.02 to 3.00, and >3.00. For the endpoints relapse-free survival and CM-specific survival, we used the Kaplan–Meier method to estimate survival probabilities. For the adjustment of potential confounders including age at diagnosis, AJCC8 stage, pT stage, tumor thickness (mm), histological type, and ulceration, we used Cox proportional hazards regression analyses. We used Fisher’s z transformation to analyze 95% confidence intervals for Spearman rank correlation coefficients. We used weighted nonparametric local regression smoothing (LOESS; smoothing factor 0.4) to graphically illustrate the association between continuous variables [18,19,20]. Statistics were carried out using the software package SAS version 9.4 (Cary, NC, USA).

## 3. Results

The entire study population consisted of 457 patients with CM, including 204 at stage I, 113 at stage II, and 140 at stage III. As shown in Table 1 and Supplementary Table S1, there were no substantial differences with respect to age and sex regarding the different pT and CM stages and healthy controls, even though PIV and NLR of CM patients were higher than in controls. As expected, tumor data (e.g., Breslow thickness) and clinical outcome (e.g., CM relapse, CM-specific survival) differed between stages (Supplementary Table S1). Interestingly, PIV and NLR values appeared to be higher in pT3 and pT4 stages when compared to pT1 and pT2 (Table 1).

**Figure 1:**
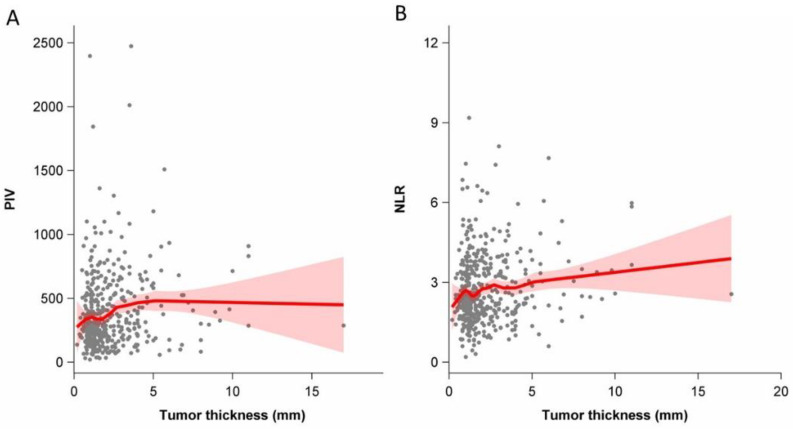
Association between tumor thickness (mm) among patients with cutaneous melanoma and the pan-immune inflammation value (PIV, (**A**)) and the neutrophil-to-lymphocyte ratio (NLR, (**B**)) (Spearman rank correlation coefficient 0.14 (95%CI 0.05–0.23) and 0.11 (95%CI 0.02–0.20), respectively); red bands indicate 95% confidence interval bands.

As shown in Figure 1A, there was a positive correlation between the PIV and tumor thickness of patients with CM (*r* = 0.14; 95% CI 0.05–0.23). Moreover, the NLR positively correlated with tumor thickness (*r* = 0.11; 95% CI 0.02–0.20; Figure 1B).

**Figure 2:**
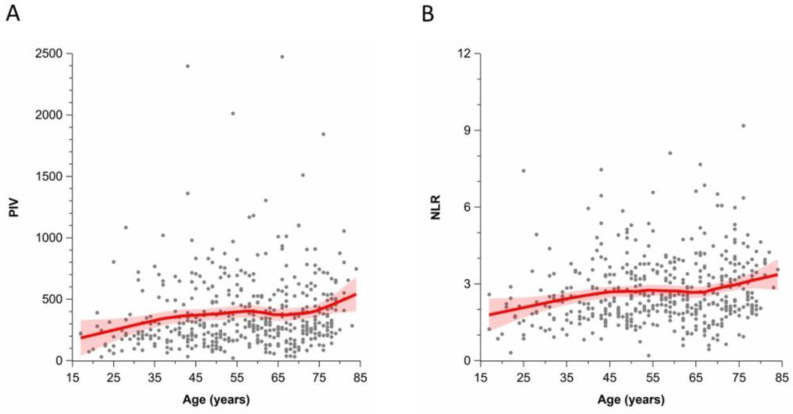
Association between age at diagnosis of patients with cutaneous melanoma and the pan-immune inflammation value (PIV, (**A**)) and the neutrophil-to-lymphocyte ratio (NLR, (**B**)) (Spearman rank correlation coefficient 0.17 (95%CI 0.08–0.26) and 0.18 (95%CI 0.09–0.27), respectively); red bands indicate 95% confidence interval bands.

As shown in Figure 2A, there was also a positive correlation between the PIV and the age of patients with CM (*r* = 0.17; 95% CI 0.08–0.26). Moreover, the NLR positively correlated with age as well (*r* = 0.18; 95% CI 0.09–0.27; Figure 2B).

**Figure 3:**
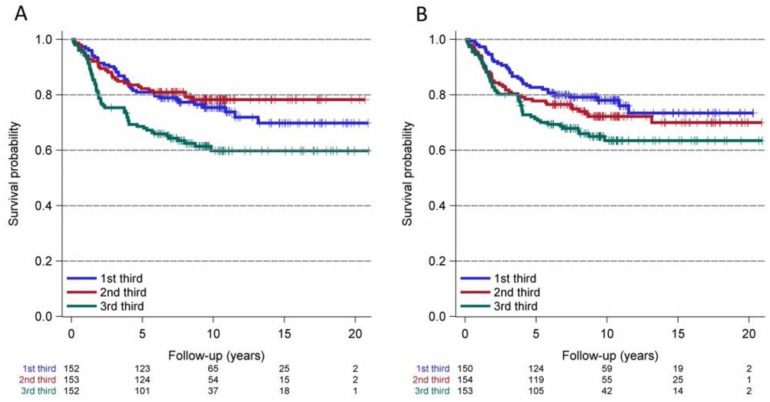
Kaplan–Meier curves for relapse-free survival of cutaneous melanoma patients and thirds of the pan-immune inflammation value (PIV, (**A**): 1st third-PIV ≤ 219.73; 2nd third-PIV > 219.73 to 393.73; 3rd third-PIV > 393.73) and the neutrophil-to-lymphocyte ratio (NLR, **B**): 1st third-NLR ≤ 2.02; 2nd third-NLR > 2.02 to 3; 3rd third-NLR > 3). 3rd thirds of PIV (**A**) and NLR (**B**) appear to be associated with poorer relapse-free survival (hazard ratio: 1.718, 95% CI 1.143 to 2.584 and hazard ratio: 1.702, 95% CI 1.104 to 2.623, respectively).

**Figure 4:**
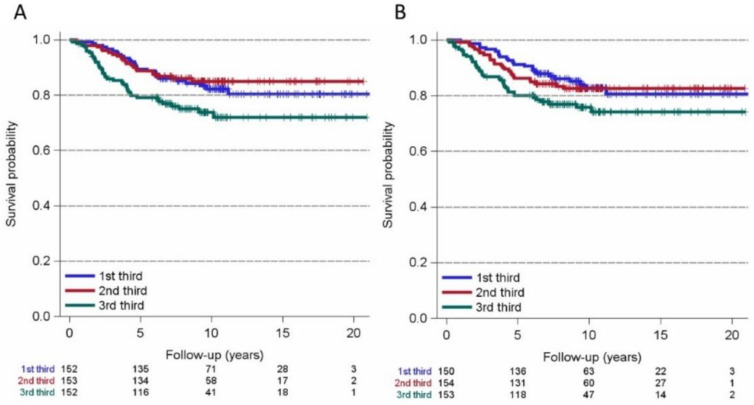
Kaplan–Meier curves for cutaneous melanoma-specific survival and thirds of the pan-immune inflammation value (PIV, (**A**): 1st third-PIV ≤ 219.73; 2nd third-PIV > 219.73 to 393.73; 3rd third-PIV > 393.73) and the neutrophil-to-lymphocyte ratio (NLR, (**B**): 1st third-NLR to 2.02; 2nd third-NLR > 2.02 ≤ 3; 3rd third-NLR > 3). The 3rd thirds of PIV (**A**) appear to be associated with poorer relapse-free survival (hazard ratio: 1.696, 95% CI 1.029 to 2.795).

For the endpoint relapse-free survival, we used the Kaplan–Meier method for PIV thirds (first PIV third ≤ 219.73; second PIV third > 219.73 to 393.73; third PIV third > 393.73) and NRL thirds (first NLR third ≤ 2.02; second NLR third > 2.02 to 3.00; third NLR third > 3.00, Figure 3). We observed that higher (third thirds) values of PIV and NLR appear to be linked to poorer relapse-free survival (hazard ratio (HR): 1.72, 95% CI 1.14 to 2.58, and HR: 1.70, 95% CI 1.10 to 2.62, respectively; Figure 3). The Kaplan–Meier curves for the endpoint CM-specific survival revealed that the third thirds of PIV also seem to be associated with poorer CM-specific survival (HR: 1.696, 95% CI 1.029 to 2.795), whereas NRL was less associated with decreased survival probability (HR: 1.6, 95% CI 0.99 to 280, Figure 4).

After adjustment for multiple confounders, including CM stage, CM subtypes, age, tumor thickness, and ulceration, the hazard ratios for the top PIV third (1.28, 95% CI 0.83 to 1.98) and NLR third (1.26, 95% CI 0.80 to 1.98) were substantially reduced with respect to CM-relapse-free survival (Table 2).

The Cox proportional hazards regression model for CM-specific survival also showed that the hazard ratios for the top PIV third (1.36, 95% CI 0.80 to 2.30) and top NLR third (1.37, 95% CI 0.80 to 2.33) were substantially reduced (Table 3).

## 4. Discussion

There is a wealth of evidence that systemic inflammatory processes play an important role in cancer development and progression [21]. Protumorigenic cytokines (e.g., vascular endothelial growth factor, tumor necrosis factor-α, interleukin-10) secreted by neutrophil granulocytes and thrombocytes may contribute to progression of malignancies [22]. Recently, systemic immune-inflammation prognosis scores were proposed to be of prognostic potential in a variety of malignancy types including CM [6,8,9,10,11,12,13,14,15,21,22,23,24,25,26,27,28,29,30]. For the first time, Fucà et al. [26] reported in 2018 on the prognostic capacity of the PIV in advanced colorectal and breast cancer. In a retrospective study on stage IV CM patients, who were managed with first-line immune checkpoint inhibitors (ICI, n = 119) or targeted therapy (n = 109), Fucà et al. [12] observed that an increase of the baseline PIV (>600) was independently linked to poor progression-free and overall survival. Moreover, they also [12] found out that an increased PIV was associated with primary resistance against ICI as well as targeted therapy. Nevertheless, that study did not investigate PIV in CM stages I to III or healthy controls. In agreement with the findings of Fuca et al. [12] who found that a higher PIV is associated with a higher M stage and increased LDH, we recently discovered that the PIV of CM patients at stage III and IV (n = 62) is higher than that of patients at stage I and II or HC [14]. Hence, the PIV could be a potential biomarker for disease burden.

Indeed, in the present study, we investigated a larger sample size of stage I to III CM patients. Surprisingly, we observed the highest median PIV and NLR in stage II patients when compared to stage I and stage III patients and HC as well (Table S1). However, when stratifying in pT 1–4 stages, there was a stepwise increase in PIV and NRL. This apparent contradiction can be explained by the fact that stage III included many patients with smaller tumor thickness (<2 mm), whereas all stage II patients had >2 mm tumor thickness. Our data clearly show that there exists a positive (albeit weak) correlation between CM thickness and PIV and NRL. Hence, PIV and NLR are mainly driven by tumor thickness and not by AJCC stages. The biology of this association is hard to explain. Indeed, it has been shown that a larger tumor load in stage IV patients is associated with higher PIV and NLR values [14,31]. However, whether the primary tumor mass is directly linked to an increase of systemic immune-inflammation markers is questionable.

Another interesting observation in this context is the fact that PIV and NLR also increase with age, whereby patients in pT4 stage were 10 years older than patients in pT1 stage. Accordingly, Fest et al. [32] assessed the prognostic potential of systemic immune-inflammatory biomarkers and found that these markers increase with age. Moreover, Li et al. [29] found that patients in the high SII (neutrophil count × platelet count/lymphocyte count) group showed poor tumor differentiation and poor prognosis compared to patients with a low SII score [29].

Multiple investigations have shown that an increased NLR is associated with poor clinical outcomes in many malignancies, but this parameter has not been thoroughly investigated in CM, except for stage IV, particularly in the context of the use of ICI [31,33,34]. Lino-Silva et al. [35] was the first study group investigating NLR in CM stages I–III. They concluded that NLR ≥ 2 seems to be a potential biomarker for diminished survival of CM patients, in particular in clinical stage II. Davis et al. [36] reported similar data for high-risk CM patients. Ma et al. [37] found similar results in CM patients at stage III. By contrast, Wade et al. [8] found that a high NLR was associated with better CM-specific survival (HR: 0.53, 95% CI 0.45 to 0.63) and disease-free survival. Exclusively considering the multivariable test data of studies on stage I to III CM patients, one study [8] demonstrated that high NLR is associated with better CM-specific survival, three studies with worse [9,28,37], and three (including the present investigation) observed no significant association between NLR and CM-specific survival [28,38]. Hence, the directionality of effect is inconsistent with respect to NLR.

Our study group systematically investigated the PIV in stage I to III CM patients for the first time. PIV appears to be higher in stage I to III CM patients when compared to healthy controls. As with our NLR data, we found associations between PIV and age, tumor thickness, CM relapse, and CM-specific death in the crude data analysis. However, when adjusting for potential confounders, in particular age and tumor thickness, the total effect of PIV on survival was substantially reduced. In fact, differences between studies are probably due to the differing inclusion criteria of previous investigations and the resulting heterogeneity of populations studied. Moreover, the utility of CBC-based systemic immune-inflammation biomarkers is also limited by other confounding factors, including inflammatory conditions, infections, or the use of immunosuppressants.

## 5. Conclusions

In a recent systematic review performed by Guven et al. [16], current evidence indicates that PIV represents an inexpensive minimally invasive prognostic tool for patients with metastatic disease. However, the present data suggest that PIV as well as NLR appear to be markedly confounded by age and tumor thickness in stage I to II CM patients. Both CBC-based parameters probably have no relevant potential in improving the prediction of survival in stage I to III CM patients.

## Figures and Tables

**Table 1:** Clinical characteristics of patients with cutaneous melanoma (CM, n = 457) in pT stages 1–4.

	Patients with CM				
Characteristics	pT_1_ (n = 94)	pT_2_ (n =196)	pT_3_ (n = 115)	pT_4_ (n = 52)	Controls (n = 49)
Males	48 (51.1)	93 (47.5)	48 (41.7)	28 (53.9)	24 (49.0)
Median Age (years; P10, P90)	54 (32; 73)	58 (34; 74)	59 (35; 77)	64 (40; 78)	61 (33;80)
CM subtype					
Superficial spreading melanoma	64 (68.1)	104 (53.1)	25 (21.7)	7 (13.5)	
Nodular melanoma	6 (6.4)	43 (21.9)	45 (39.1)	30 (57.7)	
Lentigo maligna melanoma	1 (1.1)	2 (1.0)	0	0	
Acrolentiginous melanoma	3 (3.2)	8 (4.1)	8 (7.0)	4 (7.7)	
Others	20 (21.3)	39 (19.9)	37 (32.2)	11 (21.2)	
Median tumor thickness, mm (P10, P90)	0.8 (0.5; 1.0)	1.4 (1.1; 1.8)	2.8 (2.1; 3.8)	5.5 (4.2; 9.8)	
Ulceration of primary CM, n (%)	14 (14.9)	37 (18.9)	49 (42.6)	39 (75.0)	
NLR, median (P10, P90)	2.35 (1.42; 3.86)	2.30 (1.30; 4.20)	2.53 (1.30; 4.57)	2.89 (1.56)	1.90 (1.1; 11.60)
PIV, median (P10, P90)	268 (138; 586)	269 (96; 693)	358 (121; 768)	421 (136)	276 (133; 790)
CM relapse, n (%)	9 (9.6)	39 (19.9)	47 (40.9)	33 (63.5)	
Median relapse-free survival (months) (P10, P90)	102 (18; 193)				
CM-specific death, n (%)	3 (3.2)	28 (14.3)	29 (25.2)	26 (50.0)	
Median CM-specific survival (months) (P10, P90)	110 (40; 195)				

pT: primary tumor classification; P10 10th percentile, P90: 90th percentile; SSM: superficial spreading; NLR: neutrophil/lymphocyte ratio; PIV: pan-immune inflammation value.

**Table 2:** Cox proportional hazards regression model for cutaneous melanoma (CM) relapse-free survival and the total effects for the pan-immune-inflammation value (PIV) and neutrophil-to-lymphocyte ratio (NLR) adjusted for potential confounders.

Model and Variables	*p*-Value	Hazard Ratio (HR)	95% Confidence Interval
PIV model			
PIV 2nd third (219.73 ≤ 393.73)	0.3530	0.79	0.49 to 1.29
PIV 3rd third (>393.73)	0.2689	1.28	0.83 to 1.98
Stage II	0.0950	1.69	0.91 to 3.15
Stage III	<0.0001	4.00	2.36 to 6.79
CM subtypes-ALM	0.0118	2.25	1.19 to 4.24
-LMM	0.8756	1.17	0.16 to 8.81
-NM	0.5384	1.16	0.73 to 1.85
-Others	0.8349	0.94	0.55 to 1.62
Age	0.0015	1.02	1.00 to 1.03
Tumor thickness (mm)	<0.0001	1.19	1.12 to 1.28
Ulceration	0.5755	1.13	0.74 to 1.72
NLR model			
NLR 2nd third (2.02 ≤ 3)	0.7308	1.08	0.68 to 1.73
NLR 3rd third (>3)	0.3073	1.26	0.80 to 1.98
Stage II	0.0742	1.76	0.95 to 3.29
Stage III	<0.0001	4.13	2.44 to 6.99
CM subtype-ALM	0.0093	2.30	1.23 to 4.34
-LMM	0.8679	1.19	0.16 to 8.91
-NM	0.4379	1.20	0.75 to 1.92
-Others	0.8879	0.69	0.56 to 1.65
Age	0.0010	1.02	1.01 to 1.04
Tumor thickness (mm)	<0.0001	1.18	1.11 to 1.26
Ulceration	0.5014	1.15	0.76 to 1.76

Potential confounders in gray.

**Table 3:** Cox proportional hazards regression model for cutaneous melanoma (CM)-specific survival and the total effects for the pan-immune-inflammation value (PIV) and neutrophil-to-lymphocyte ratio (NLR) adjusted for potential confounders.

Model and Variables	*p*-Value	Hazard Ratio (HR)	95% Confidence Interval
PIV model			
PIV 2nd third (219.73 ≤ 393.73)	0.6476	0.87	0.48 to 1.57
PIV 3rd third (>393.73)	0.2553	1.36	0.80 to 2.30
Stage II	0.2159	1.66	0.75 to 3.68
Stage III	0.0002	3.72	1.85 to 7.46
CM subtype-ALM	0.7185	1.17	0.49 to 2.80
-LMM	0.5596	1.83	0.24 to 14.11
-NM	0.8061	1.07	0.61 to 1.88
-Others	0.2259	0.64	0.31 to 1.31
Age	0.0099	1.02	1.00 to 1.04
pT (1–4)	<0.0001	1.17	1.08 to 1.26
Ulceration	0.0180	1.84	1.11 to 3.04
NLR model			
NLR 2nd third (2.02 ≤ 3)	0.7537	0.91	0.51 to 1.62
NLR 3rd third (>3)	0.2543	1.37	0.80 to 2.33
Stage II	0.2239	1.64	0.74 to 3.66
Stage III	0.0002	3.79	1.90 to 7.57
CM subtypes-ALM	0.6600	1.21	0.51 to 2.88
-LMM	0.4884	2.06	0.27 to 15.87
-NM	0.6541	1.14	0.65 to 1.99
-Others	0.2472	0.66	0.32 to 1.34
Age	0.0089	1.02	1.00 to 1.04
pT (1–4)	<0.0001	1.16	1.08 to 1.25
Ulceration	0.0145	1.87	1.13 to 3.09

Potential confounders in gray.

## Data Availability

Derived data supporting the findings of this study are available from the corresponding author on reasonable request.

## Supplementary Materials

The following supporting information can be downloaded at: https://www.mdpi.com/article/10.3390/cancers14184410/s1, Table S1: Clinical characteristics of patients with cutaneous melanoma (CM, n = 457) in AJCC (8th edition) stages I–III and healthy controls (n = 49).
