# Supplementary material for: Prognostic Potential of the Baseline Pan-Immune-Inflammation Value and Neutrophil/Lymphocyte Ratio in Stage I to III Melanoma Patients
Source: Cancers (Basel). 2022 Sep 11;14(18):4410. doi: 10.3390/cancers14184410 (PMC9496745; doi:10.3390/cancers14184410)
Supplement: Supplementary file 1 [file cancers-14-04410-s001.zip › cancers-1721976-supplementary.pdf]

Supplementary Materials：Prognostic Potential of the Baseline Pan-Immune-Inflammation Value and Neutrophil-to-Lymphocyte Ratio in Stage I to III Melanoma Patients

Table S1. Clinical characteristics of patients with cutaneous melanoma (CM, n=457) in AJCC (8th edition) stages I-III and healthy controls (n=49).

	Patients with CM				Controls (n=49)
**Characteristics**	All (n=457)	AJCC I (n=204)	AJCC II (n=113)	AJCC III (n=140)	
**Males**	217 (47.5)	94 (46.1)	48 (42.3)	75 (53.6)	24 (49.0)
**Median Age** (P10, P90)	58 (34; 76)	57 (33; 74)	62 (35; 76)	56 (36.5; 77.5)	61 (33; 80)
**CM subtype**					
Superficial spreading melanoma	200 (43.8)	129 (63.2)	31 (27.4)	40 (28.6)	
Nodular melanoma	124 (27.1)	28 (13.7)	37 (32.7)	59 (42.1)	
Lentigo maligna melanoma	3 (0.7)	1 (0.5)	1 (0.9)	1 (0.7)	
Acrolentiginous melanoma	23 (5.0)	7 (3.4)	9 (8.0)	7 (5.0)	
Others*	107 (23.4)	39 (19.1)	35 (31.0)	33 (23.6)	
**Median tumor thickness**, mm (P10, P90)	1.6 (0.8, 4.3)	1.1 (0.7; 1.6)	2.6 (1.5; 5.5)	2.4 (1.1; 5.6)	
**Ulceration of primary CM**, n (%)	139 (30.4)	12 (5.9)	69 (61.1)	58 (41.4)	
**NLR, median** (P10, P90)	2.41 (1.36; 4.28)	2.31 (1.36; 4.24)	2.63 (1.30; 4.74)	2.44 (1.39; 4.08)	1.90 (1.10; 11.60)
**PIV, median** (P10, P90)	291 (118; 715)	277 (119; 688)	364 (121; 861)	291 (115; 662)	276 (133; 790)
**CM relapse**, n (%)	128 (28.0)	23 (11.3)	37 (32.7)	68 (48.6)	
**Median relapse-free survival** (P10, P90)	102 (18; 193)				
**CM-specific death**, n (%)	86 (18.8)	13 (6.4)	26 (23.0)	47 (33.6)	
**Median CM-specific survival** (P10, P90)	110 (40; 195)				

AJCC: American Joint Cancer Committee; P10 10th percentile, P90: 90th percentile; *others including unclassified melanoma; NLR: neutrophil/lymphocyte ratio; PIV: pan-immune inflammation value.
